# Assessment to determine the accuracy of Chaillet and Demirjian method of dental age estimation using Orthopantomographs, Eastern Province, Saudi Arabia

**DOI:** 10.12688/f1000research.157275.2

**Published:** 2025-03-06

**Authors:** Faraz Mohammed, Arishiya Thapasum Fairozekhan, Intisar Ahmad Siddiqui, Saud AlMoumen, Turki Ali AlShehri, Maria Hassan AlRssasi, Abdullah Amer AlJami, Doha Mohsen AlRamadan, Ashwin C Shetty, Majed Mohammed Alfarea, Omar Suhaym, Nasser S AlShahrani

**Affiliations:** 1 Department of Biomedical Dental Sciences, College of Dentistry, Imam Abdulrahman Bin Faisal University, Dammam, Saudi Arabia; 2 College of Dentistry, Imam Abdulrahman Bin Faisal University, Dammam, Saudi Arabia; 3 Department of Dental Education, College of Dentistry, Imam Abdulrahman Bin Faisal University, Dammam, Saudi Arabia; 4 King Abdullah International Medical Research Center, Riyadh, Riyadh Province, Saudi Arabia; 5 King Saud bin Abdulaziz University for Health Sciences, Riyadh, Riyadh Province, Saudi Arabia

**Keywords:** forensic science; age estimation; Demirjian method; dental age; chronological age

## Abstract

**Background:**

The approach to estimating mandibular age has been widely used, although it has shown age estimation disparities in diverse populations, including Indians.

**Objective:**

Our goal was to test the Demirjian 8-teeth approach utilizing orthopantomogram to estimate age reliably after incorporating the third molar. We also used Chaillet and Demirjian’s regression equations to estimate age estimation accuracy in the Eastern Province of Saudi Arabia.

**Methods:**

The retrospective study included 196 people. Individuals were 8–24 years old. The left mandibular teeth were staged using an orthopantomographs utilizing the Demirjian 8-teeth approach. The Chaillet and Demirjian’s regression models determined dental age, and statistical methods compared dental age to chronological age.

**Results:**

The gender breakdown was 49.5% male and 50.5% female. Gender did not significantly affect chronological mean age or estimated mean age (13.39±3.77 vs. 13.10±3.51, p = 0.583; 11.75±2.92 vs. 11.58±2.70, p = 0.674). Statistically substantial differences in chronological and estimated mean ages between male and female individuals (p<0.001). Out of the total participants, 31.6% had a difference in age of ±1 year, 30.6% had a difference of 1-2 years, and 37.8% had a difference of more than 2 years. Compared to chronological and estimated ages, these variations in age between males and females were not statistically significant (p=0.557).

**Conclusions:**

The findings of this study support the use of Demirjian’s 8-teeth approach in the Saudi population residing in the Eastern Province of the Kingdom of Saudi Arabia, employing Chaillet and Demirjian’s regression equations.

## Introduction

The current emphasis in forensic science and medico-legal studies revolves around the crucial significance of forensic age estimation. ^1, 2^ Forensic dental age estimation is well recognized as a very precise and established technique employed in the determination of the chronological age of individuals involved in judicial or legal processes. ^1^ The determination of age in children and teenagers is again a crucial aspect in addressing a wide range of legal investigations. In the present context, the majority of age estimation methods are invasive in nature, necessitating extended processing durations, utilization of costly equipment, and the expertise of a skilled pathologist to infer the age of an individual. ^3^ The growth and eruption of dentition are significant factors in determining dental age and are widely regarded as the most reliable physiological non-invasive indicators for measuring chronological age in children and juveniles. ^4– 6^ The process of tooth mineralization is comparatively less influenced by nutritional intake, endocrine status, and local conditions when compared to the process of bone mineralization.

Demirjian et al. proposed a methodology for age estimation that relied on the analysis of seven mandibular teeth. The methodology has been extensively utilized; however, it has demonstrated discrepancies in age estimations among a limited number of populations. The observed variations in tooth eruption timing among different ethnicities worldwide have been attributed to racial differences. The assessment of age based on tooth development is subject to the influence of two primary elements: genetic variability and environmental factors. ^4^ Multiple countries have made various efforts to minimize the influence of variability in dental age estimation. This is achieved by establishing genetically similar populations and developing standardized tables that are specifically applicable to these genetically similar individuals. ^2, 7, 8^ Consequently, it is crucial to derive dental age estimates from the population in which they will be utilized. Additionally, one limitation of the first Demirjian et al. approach was its exclusion of the third molar due to its potential for congenital absence and the significant variability in its development. ^9^ Demirjian et al. conducted a study in which they categorized the progression of tooth growth into eight distinct stages and subsequently devised a method for estimating age based on these stages.

Certain researchers observed a notable level of precision within their examined sample, while others documented instances of either overestimating or underestimating dental age in relation to chronological age. ^10^ Yet another study found that the Chaillet and Demirjian method underestimated the dental age of Malaysian Chinese individuals. ^11^

Thus, the primary objectives of our study were to validate the Demirjian 8-teeth method using orthopantomogram for age estimation, specifically by incorporating the third molar, and to assess the accuracy of age estimation in the Saudi population from the Eastern Province of the Kingdom of Saudi Arabia using Chaillet and Demirjian’s regression formulae.

## Materials and methods

The present study employed materials and methods approach to investigate the research question.

The present investigation was undertaken at the Dental College Hospital of Imam Abdulrahman bin Faisal University (IAUDent) in Dammam, Kingdom of Saudi Arabia. The present retrospective investigation was conducted over a duration of one year, utilizing archived orthopantomograms (OPGs) that were originally collected for diagnostic and therapy purposes. The age, gender, and date of image acquisition for patients were obtained from the orthopantomography system’s records within the hospital.

The present study involved a retrospective, cross-sectional design and focused on a specific sample of orthopantomograms (OPGs) obtained from 196 individuals who were of Eastern Province origin in the Kingdom of Saudi Arabia. The subjects chosen for the study fell within the age bracket of 8 to 24 years. The OPGs and associated data were acquired from the database archives of the centrally stored radiology computer software MiPACS at Imam Abdulrahman bin Faisal University’s Dental College Hospital (IAUDent), Dammam, Kingdom of Saudi Arabia. This data collection occurred between October 2023 and December 2023, following a waiver of ethical approval granted by the institutional review board of Imam Abdulrahman bin Faisal University on October 10, 2023.

The study comprised patients who had a complete set of maxillary and mandibular right and left quadrant teeth, with no impacted, embedded, or trans-positioned teeth, and whose Orthopantomograms (OPGs) were free of any position errors or artifacts. Subjects who exhibited any pathological conditions, had previously undergone tooth extraction or restorative procedures, were currently undergoing orthodontic treatment, had congenital or developmental anomalies of the jaws and teeth, or had a history of craniofacial trauma, jaw disorders, or systemic illnesses were excluded from the study. The exclusion criteria were verified by cross-referencing the subjects’ reception files. Table 1 displays the gender-based distribution of subjects. The age, date of birth, gender, and nationality of the subject were extracted from the patients’ case file records.

**Table 1.** Distribution of subjects by gender.

	Gender	Frequency	Percent	Valid percent	Cumulative percent
Valid	Male	97	49.5	49.5	100.0
	Female	99	50.5	50.5	50.5
	Total	196	100.0	100.0	

### Calculation of chronological age (CA)

The verification of the Date of Birth (DOB) was conducted by cross-referencing the information with the official birth document that was recorded during the registration process at the IAUDent Hospital. The Date of Exposure of Radiograph (DOR) was obtained from the MiPACS system. The calculation of chronological age in decimal years involved subtracting the date of birth (DOB) from the date of reference (DOR) using a straightforward formula in Microsoft Excel: (DOR-DOB/365.25).

### Calculation of Dental Age (DA) using Demirjian’s method

The dental age (DA) can be determined with the application of Demirjian’s method ^12^ for calculation. The Chaillet and Demirjian regression formulae were selected for this study because they are widely recognized and validated methods for dental age estimation based on orthopantomographs (OPGs). These formulae, derived from Demirjian’s original methodology, have been frequently used in forensic and anthropological studies due to their reliability and applicability across various populations globally. Given that dental development patterns can vary among racial groups, this study was aimed to validate the accuracy of the Chaillet and Demirjian regression formulae specifically for these ethnically homogeneous individuals of Saudi Arabian origin from the Eastern Province. Further the validation of this established regression model in a new population was scientifically warranted due to the lack of population-specific dental age estimation models in the Eastern Province of kingdom of Saudi Arabia.

The dental structures located on the left side of the mandible, specifically ranging from the mandibular central incisor to the mandibular third molar, were assessed using Demirjian’s modified criteria. This evaluation involved the utilization of 10 distinct stages of tooth development. In cases when a tooth is absent in the mandibular left quadrant, the corresponding tooth on the contralateral side was included as part of the research investigation. The dental age was determined by employing the formula outlined in the method proposed by Chaillet and Demirjian, utilizing their regression equations. ^12^

The equation for determining the age of males is as follows:

$$\mathrm{Age}=\left(0.000055\;\times \;S^{3}\right)-\left(0.0095\;\times \;S^{2}\right)+\left(0.6479\;\times \;S\right)–8.4583.$$

The equation to estimate the age of females is given by

$$\mathrm{Age}=\left(0.0000615\;\times \;S^{3}\right)–\left(0.0106\;\times \;S^{2}\right)+\left(0.6997\;\times \;S\right)–9.3178,$$

where S represents a specific variable.

The dental age estimation was performed by two independent observers (FM & ATF), both of whom were qualified Oral Pathologist and Oral Radiologist respectively with extensive experience in age estimation techniques. The interobserver agreement for the first observer (FM) yielded a kappa value of 0.907, indicating a strong level of agreement. Similarly, the second observer (ATF) achieved a kappa value of 0.911, also indicating a strong level of agreement. The interobserver agreement between examiners FM and ATF were found to be 0.882, reflecting a substantial correlation. Any discrepancies in age estimation between the two observers were resolved through discussion and consensus.

### Statistical analysis

The data collected, which included Chronological Age (CA), and Dental Age (DA) computed using the Demirjian technique, were inputted into the Statistical Package for Social Sciences (IBM Product, SPSS Version-25.0, USA). The mean and standard deviation were calculated for the numeric variables representing the chronological and predicted age of the individuals. The statistical analysis employed in this study was the use of a paired sample t-test to compare the differences between chronological age and estimated age for both the whole sample and for individual gender groups. The categorization of the disparities between chronological and estimated age was conducted based on three intervals: ±1 year, ±>1-2 years, and ±>2 years. The chi-square test was utilized to compare the categorized age differences based on combination and gender variables. A significance level of P < 0.05 was deemed to indicate statistical significance.

## Results

**Figure 1.**
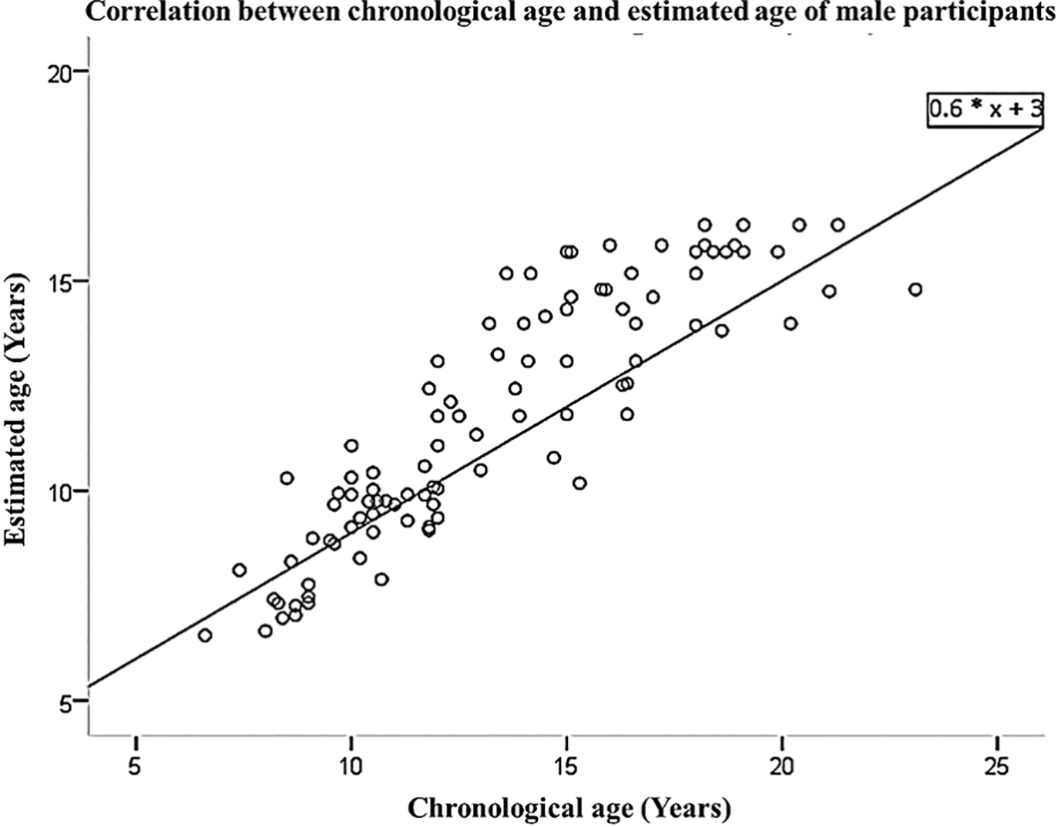
Correlation between chronological age and estimated age of male participants.

**Figure 2.**
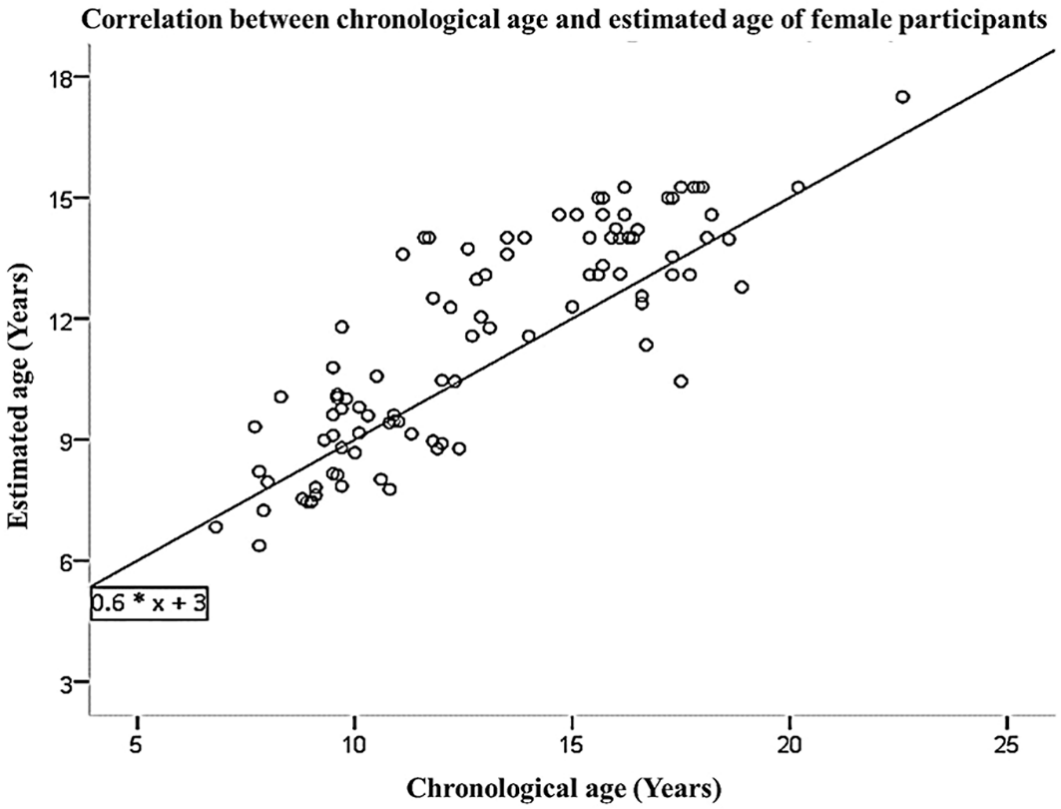
Correlation between chronological age and estimated age of female participants.

The study involved the evaluation of 196 panoramic radiographs obtained from a sample of 99 male and 97 female participants, all within the age range of 8 to 24 years. Table 1 illustrates a gender distribution that is evenly split, with 49.5% of individuals identified as men and 50.5% identified as females. The results indicate that there were no significant differences in the chronological mean age and estimated mean age based on formula between male and female participants. Specifically, the mean ages for males and females were 13.39±3.77 and 13.10±3.51, respectively, with a p-value of 0.583. Similarly, the mean ages based on formula were 11.75±2.92 for males and 11.58±2.70 for females, with a p-value of 0.674. However, when considering the overall differences between male and female participants’ chronological mean age and estimated mean age, there was a statistically significant difference (p<0.001), as shown in Table 2. The study found that 31.6% of subjects had an age difference of ±1 year, while 30.6% had an age difference of ±>1-2 years, and 37.8% had an age difference greater than 2 years. Nevertheless, the observed disparities in chronological and estimated ages did not reach statistical significance, as seen in Table 3. The linear regression equation, which was derived from the data collected from both male and female participants, allows for the prediction of chronological age based on estimated age. This equation is represented as Y=3+0.6*X, where X and Y represent the estimated and predicted chronological ages, respectively. These findings are visually presented in Figures 1 and 2. The interobserver agreement for the first examiner (FM) yielded a Kappa value of 0.872, indicating a strong level of agreement. Similarly, the second examiner (ATFK) achieved a Kappa value of 0.838, also indicating a strong level of agreement. The interobserver agreement among examiners regarding FM and ATFK, the value was determined to be 0.812, indicating a substantial correlation. The analysis of means did not yield statistically significant differences, indicating that the current age estimation equation can be applied to estimate the age of individuals within the Saudi population, taking into account gender-specific considerations.

**Table 2.** Mean difference of chronological age versus estimated age.

Gender		Estimated age	Chronological age	Difference of age	Significance
Male	Mean	11.75±2.92	13.38±3.77	1.630	0.674
	Std. Deviation	2.97	3.75	1.786	
Female	Mean	11.502±2.70	13.10±3.51	1.521	0.583
	Std. Deviation	2.74	3.54	1.848	
Total	Mean	11.689±2.81	13.24±3.63	1.579	<0.001
	Std. Deviation	2.89	3.69	1.814	

**Table 3.** Difference of chronological age versus estimated age.

Difference of chronological vs. Estimated age	± 1 year	±>1–2 years	±>2 years	Significance
Male	33 (34.0)	31 (32.0)	33 (34.0)	0.961
Female	29 (29.3)	29 (29.3)	41 (41.4)	0.234
Combined	62 (31.6)	60 (30.6)	74 (37.8)	0.653

## Discussion

The utilization of tooth structures as a means of estimating age has been a widely employed practice with a long-standing history. Edwin Saunders, in the year 1837, made a significant contribution to the field of age assessment by presenting a pamphlet titled “Teeth A Test of Age” to the English Parliament. ^13^ Subsequently, other approaches for estimating age have been suggested; yet, regrettably, a universally applicable method remains elusive. The absence of this trait can be ascribed to the substantial degree of variance observed among many ethnic groups and populations. ^14^ Demirjian’s method is a widely employed approach for age estimation due to its simplicity, practicality, and ease of comprehension. ^13^ Throughout history, other scholars have also conducted experiments to assess the validity of this formula in diverse communities and have suggested alterations to this methodology. Chaillet-Demirjian incorporated the inclusion of the third molar into the initial formula utilized for evaluating the age of French children, afterwards deriving regression formulas for age assessment. ^12^ A significant alteration implemented in this study involved the incorporation of two supplementary steps in the process of tooth staging. This adjustment was done to facilitate the calculation process and to establish cubic equations that exhibit a high degree of reliability. ^15^ Demirjian’s method is characterized by its well-defined criteria, which effectively eliminates the need for speculative estimation. Consequently, this approach is highly comprehensible and replicable. The methodology employed in this study relies on evaluating the level of calcification in dental structures, namely until the point of root apex closure, while also considering the proportional lengths of the crown and root.

The Kingdom of Saudi Arabia is currently seeing a growing need for age estimation in both children and adults. This demand is primarily driven by many factors like human trafficking, migration, asylum procedures, child pornography, adoption of children without proper birth certificates, and legal considerations, among others. ^16^ Multiple research studies have been conducted on the Saudi and international population, which have identified a tendency to overestimate age when utilizing Demirjian’s age estimation approach. It is important to acknowledge that the earlier investigations employed Demirjian’s 7-teeth method, while the current study employed the modified 8-teeth method. There exists a limited number of studies that have investigated the precision of the Chaillet-Demirjian age estimation method. The Chaillet-Demirjian’s method demonstrates minimal disparity between predicted age and chronological age when all seven complementary teeth are present, making it a viable technique for age estimation. Moreover, the method proposed by Chaillet and Demirjian has undergone validation using reference data relevant to multiple countries. ^17^ Nevertheless, the efficacy of this approach in estimating age among the Saudi population residing in the Eastern province has not yet been investigated. Consequently, it remains uncertain if a region-specific reference technique is warranted for individuals from the Eastern province of Saudi Arabia. Hence, the primary objective of this study was to validate the regression formulas approach proposed by Chaillet and Demirjian in a sample of individuals from the Eastern province of Saudi Arabia. Additionally, this study aimed to propose the development of region-specific formulae and a method for age estimation in the Eastern province of Saudi Arabia, if deemed necessary.

The data was obtained from the Dental College Hospital (IAUDent) at Imam Abdulrahman bin Faisal University in Dammam, Kingdom of Saudi Arabia. This institution is highly regarded in the region and is representative of the entire population of the Eastern province in the Kingdom. The mean difference between the estimated and chronological ages in the entire sample of 196 orthopantomograms was found to be 1.57±0.82 years, indicating a statistically significant difference (p<0.001), as shown in Table 2. In males, the mean difference was found to be 1.63±0.85 years, whereas in females, it was seen to be 1.52±0.81 years. The observed differences were determined to be statistically nonsignificant.

Numerous research has been undertaken across diverse populations with the regression equations method developed by Chaillet and Demirjian, yielding varying outcomes. Certain researchers observed a notable level of precision within their examined sample, while others documented instances of either overestimating or underestimating dental age in relation to chronological age. Numerous investigations, including the present investigation, have identified a tendency to underestimate estimated age relative to chronological age. In a study conducted by AlOtaibi and AlQahtani in 2023, it was observed that there was an underestimation of -2.03 years in males and -2.35 years in females throughout the Saudi population across all age categories. ^18^ A notable disparity between dental age and chronological age was noticed among the male and female individuals of the Belgian Caucasian community. The combined mean absolute error for both males and females was found to be 1.57 years, with only 31.6% of cases accurately predicted to fall within a range of 1 year of their chronological age. A statistically significant difference was observed, suggesting that Chaillet and Demirjian’s study on the Belgian Caucasian population did not demonstrate a strong association with the population of the Eastern Province in Saudi Arabia. Therefore, it is necessary to utilize population-specific weighted scores when applying the values proposed by Chaillet and Demirjian to this particular population. The variation observed between estimated and chronological ages in various studies, including the present one, can be attributed to the inherent diversity found in ethnicity, culture, sample size, environmental factors, socioeconomic status, nutrition, dietary habits, statistical methods, and subjectivity of the examiner across different populations. The study also revealed that the phenomenon of age underestimation is less prevalent among females compared to males. This discovery suggests that girls reach maturity at an earlier stage compared to males, which aligns with the observed early maturation of skeletal age in females. The observations suggest that girls in Saudi Arabia have a higher level of dental maturity in comparison to boys, which aligns with findings from previous studies.

### Scope for improvement

The authors want to broaden their research scope by establishing cubic regression formulae that are specific to the population of the Eastern province in the Kingdom of Saudi Arabia. This expansion is motivated by the recognition of growth variances across different populations, which can be attributed to distinct genetic and environmental factors. It is recommended that the maturity scores accurately reflect the specific population under examination. Consequently, it is suggested that the utilization of weighted scores for the Eastern province be pursued as a more favorable approach for evaluating dental age in future research endeavors.

## Conclusions

In light of the evidence presented, it can be inferred that the findings support the stated hypotheses. The primary objective of this study was to assess the suitability and precision of the Chaillet and Demirjian’s regression equations in predicting the age of individuals within the population of Saudi Arabia’s Eastern province. This study was characterized by two notable aspects: the utilization of digital radiographs for analysis and the incorporation of a larger sample size encompassing individuals aged 8 to 24 years. Based on the findings of this study, it can be inferred that the regression formulae developed by Chaillet and Demirjian may not be sufficiently applicable to the population of the Eastern Province in Saudi Arabia, as the average age difference observed was approximately two years. Nevertheless, for the purpose of achieving a more accurate estimation of age, it would be more advantageous to employ a gender-specific approach in the implementation of this strategy. Hence, it is imperative to establish a distinct set of equations and methodologies for age estimate among individuals of Saudi Arabian Eastern province origin, rather than relying on the Chaillet and Demirjian’s reference database.

## Ethical approval

This data collection occurred between October 2023 and December 2023, following a waiver of ethical approval granted by the institutional review board of Imam Abdulrahman Bin Faisal University on October 10, 2023.

The ethical approval was waived for this study because it was retrospective, and no direct involvement or interaction with human participants occurred. The study utilized previously collected data, which ensured that no additional risks were posed to individuals.

## Consent

Patients’ written and informed consent was taken when they received treatment in the hospital. This study used X-rays retrospectively. Hence, the statement was not mentioned in the manuscript.

## Data availability statement

Figshare: Assessment to determine the accuracy of Chaillet and Demirjian method of dental age estimation using Orthopantomographs, Eastern Province, Saudi Arabia. DOI: https://doi.org/10.6084/m9.figshare.27129798.v1. ^19^

The project contains the following underlying data:

• Data.xlsx

Data are available under the terms of the Creative Commons Attribution 4.0 International license (CC-BY 4.0).
