# Supplementary material for: Assessment to determine the accuracy of Chaillet and Demirjian method of dental age estimation using Orthopantomographs, Eastern Province, Saudi Arabia
Source: F1000Res. 2025 Mar 6;13:1554. Originally published 2024 Dec 23. [Version 2] doi: 10.12688/f1000research.157275.2 (PMC11890266; doi:10.12688/f1000research.157275.2)
Supplement: Peer review history

# Reviewer report: Reviewer response for version 2

**DOI:** 10.5256/f1000research.178533.r369811

**Published:** 2025-03-08

**Authors:** Dwi Kartika Apriyono

**Affiliations:** 1 University of Jember, Jember, Indonesia

In my opinion, the revisions made by the authors are sufficient to answer my question so it can proceed to the next stage.

Is the work clearly and accurately presented and does it cite the current literature?

Partly

If applicable, is the statistical analysis and its interpretation appropriate?

Yes

Are all the source data underlying the results available to ensure full reproducibility?

Yes

Is the study design appropriate and is the work technically sound?

Yes

Are the conclusions drawn adequately supported by the results?

Yes

Are sufficient details of methods and analysis provided to allow replication by others?

Yes

Reviewer Expertise:

forensic odontology

I confirm that I have read this submission and believe that I have an appropriate level of expertise to confirm that it is of an acceptable scientific standard.

# Reviewer report: Reviewer response for version 1

**DOI:** 10.5256/f1000research.172700.r364859

**Published:** 2025-02-07

**Authors:** Dwi Kartika Apriyono

**Affiliations:** 1 University of Jember, Jember, Indonesia

Some things that need to be fixed or explained are:

 1. The reasons for choosing the Chaillet and Demirjian's regression formulae have not been stated.

 2. it is necessary to add the racial category of the research subjects

 3. It is necessary to convey the number of observers and the frequency of calculating dental age.

 4. Need to add up-to-date literature

Is the work clearly and accurately presented and does it cite the current literature?

Partly

If applicable, is the statistical analysis and its interpretation appropriate?

Yes

Are all the source data underlying the results available to ensure full reproducibility?

Yes

Is the study design appropriate and is the work technically sound?

Yes

Are the conclusions drawn adequately supported by the results?

Yes

Are sufficient details of methods and analysis provided to allow replication by others?

Yes

Reviewer Expertise:

forensic odontology

I confirm that I have read this submission and believe that I have an appropriate level of expertise to confirm that it is of an acceptable scientific standard, however I have significant reservations, as outlined above.

# Response

**Published:** 2025-02-10

**Authors:** Ashwin Shetty

**Affiliations:** Dental Education, Imam Abdulrahman Bin Faisal University, Dammam, Eastern Province, Saudi Arabia

Dear Reviewer,

 Thank you for your time in reviewing our article. Your insightful comments mean a lot to us. We have carefully revised the manuscript to address your concerns, and we appreciate your valuable feedback in improving our work.

 Thanks 

 Authors

# Response

**Published:** 2025-02-20

**Authors:** Ashwin Shetty

**Affiliations:** Dental Education, Imam Abdulrahman Bin Faisal University, Dammam, Eastern Province, Saudi Arabia

Dear Reviewer, 

 Thank you for your valuable comments. 

 Please find below the point-by-point response to your comments. 

**1. The reasons for choosing Chaillet and Demirjian's regression formulas have not been stated.**

 Response: Thank you. The reason for choosing Chaillet and Demirjian's regression is added in the method section. 

**2. It is necessary to add the racial category of the research subjects.**

 Response: The participants of this study were ethnically homogeneous, comprising individuals of Saudi Arabian origin from the Eastern Province. Thank you.

**3. It is necessary to convey the number of observers and the frequency of calculating dental age.**

 Response: Details on the observers are added in the method section in the revised version. Thank you.

**4. Need to add up-to-date literature**

 Response: Thank you for your comment. The up-to-date literature is added in the updated version.

# Reviewer report: Reviewer response for version 1

**DOI:** 10.5256/f1000research.172700.r352324

**Published:** 2025-01-06

**Authors:** Shaul Hameed Kolarkodi

**Affiliations:** 1 Qassim university, Qassim, Saudi Arabia

- The current research article reflects its originality, best presentation, and relevance for the readers. The author's message is appropriate.
- Major strengths include comprehensive and rigorous methodology which ensures a thorough analysis, and the authors discussed in detail.
- The scientific content is high with good English language this paper will contribute to the scientific literature.

Is the work clearly and accurately presented and does it cite the current literature?

Yes

If applicable, is the statistical analysis and its interpretation appropriate?

Yes

Are all the source data underlying the results available to ensure full reproducibility?

Yes

Is the study design appropriate and is the work technically sound?

Yes

Are the conclusions drawn adequately supported by the results?

Yes

Are sufficient details of methods and analysis provided to allow replication by others?

Yes

Reviewer Expertise:

forensic odontology. age estimation, and oral cancer. premalignant lesions and conditions.

I confirm that I have read this submission and believe that I have an appropriate level of expertise to confirm that it is of an acceptable scientific standard.

# Response

**Published:** 2025-02-10

**Authors:** Ashwin Shetty

**Affiliations:** Dental Education, Imam Abdulrahman Bin Faisal University, Dammam, Eastern Province, Saudi Arabia

Dear Reviewer,

 Thank you for your time in reviewing our article. Your comments mean a lot to us and will help improve our work.
